# Invasive cutaneous *Neoscytalidium* infections in renal transplant recipients: a series of five cases

**DOI:** 10.1186/s12879-015-1241-0

**Published:** 2015-11-19

**Authors:** Simon Garinet, Jérôme Tourret, Stéphane Barete, Nadia Arzouk, Isabelle Meyer, Camille Frances, Annick Datry, Dominique Mazier, Benoit Barrou, Arnaud Fekkar

**Affiliations:** AP-HP, Groupe hospitalier La Pitié-Salpêtrière, Service de Parasitologie Mycologie, F-75013 Paris, France; Département d’urologie, néphrologie et transplantation, AP-HP, Groupe hospitalier Pitié-Salpêtrière, F-75013 Paris, France; Sorbonne Universités, UPMC Univ Paris 06, F-75005 Paris, France; AP-HP, Groupe hospitalier Pitié-Salpêtrière, Unité fonctionnelle de Dermatologie F-75013, Université Paris Sorbonne-UPMC, Paris, France; Centre d’Immunologie et des Maladies Infectieuses, CIMI-Paris, F-75013 Paris, France

**Keywords:** Fungal infection, Renal transplant, Solid organ transplant, Immunodepression, Moulds

## Abstract

**Background:**

*Neoscytalidium species* (formerly *Scytalidium species*) are black fungi that usually cause cutaneous infections mimicking dermatophytes lesions. Very few publications have reported invasive or disseminated infections.

**Case presentation:**

In this paper, we report the clinical presentations, treatments and outcomes of five cases of invasive *Neoscytalidium* infections with cutaneous involvement, including two cases with disseminated infection, in five renal transplant recipients. To our knowledge, this is the first report of a series—albeit small—of renal transplant patients in whom this infection was identified. All cases occurred in a single hospital in Paris, France, between 2001 and 2011. Patients all originate from tropical area.

**Conclusion:**

Treatments of *Neoscytalidium* infection varied greatly, underlining the lack of a recommendation for a standardized treatment. All patients were cured after long-term antifungal therapy and/or surgical excision. Interestingly, one patient with disseminated infection involving the left elbow, the right leg, the lungs and the nasal septum was cured by medical therapy only without surgery. This may suggest that in contrast to others mycoses (such as mucormycosis), an adequate medical treatment could be sufficient for treating *Neoscytalidium*. We also point out the difficulties we had in diagnosing two patients with Kaposi’s sarcoma because of the similarity of the lesions. Furthermore, our report underlines the need to check for this rare infection in immunocompromised kidney transplant recipients originating from tropical areas.

## Background

*Neoscytalidium* spp. (formerly *Scytalidium*) are ascomycetous fungi counted among a large and heterogeneous group of fungi that cause phaeohyphomycosis. One of the particular characteristics of these fungi, known collectively as dematiaceous fungi or black mold, is to produce dark melanized hyphae [1]. They are distributed worldwide in tropical and subtropical regions and are principally known as phytopathogens [1–4]. Thus, they are mainly associated with plants and fruit trees, but can also be isolated from soil [1, 5].

Some however can infect humans via direct contact, sometimes traumatic, with colonized plants or vegetable residues [1]. They usually cause superficial infections such as onychomycosis or dermatomycosis and are considered as “dermatophytes-like” pathogens [1–6]. The two species involved in human infections are *Neoscytalidium dimidiatum* and its albino variant *Neoscytalidium hyalinum*, which does not produce melanin [1, 7]. *N. dimidiatum* is the synanamorph of *Nattrassia mangiferae* (formerly *Hendersonula toruloidea*) that was described for the first time in Egypt in 1933 by Nattrass as a phytopathogen [8]. The first cases in human infection were described in 1970 and consisted of ungual and cutaneous lesions in patients originating from tropical areas [9]. The first case of *N. hyalinum* was described a few years later in patients with similar lesions [10].

On rare occasions, *Neoscytalidium* may be involved in deeper cutaneous and subcutaneous infections, as well as disseminated infections, especially in immunocompromised patients. In this setting, various clinical forms have been described, including central nervous system abscesses, endophthalmitis, sinusitis, osteomyelitis, mycetoma, subcutaneous lesions and disseminated infections [1, 11–21].

We report here the clinical presentations, treatments and outcomes of five deep cutaneous infections due to *Neoscytalidium* spp*.*, two of which involved dissemination, that occurred in five renal transplant recipients in our institution in Paris, France.

## Materials and methods

### Definition of the cases

Proven invasive fungal disease was as defined by the EORTC/MSG Consensus Group [22]. An invasive case was defined as the presence of fungal elements in a specimen obtained by biopsy or needle aspiration from a normally sterile site. Dissemination was defined as the involvement of two or more non-contiguous sites.

### Culture

Cultures for all samples were performed on Sabouraud agar without cycloheximide and supplemented with chloramphenicol and gentamicin. Media were incubated at 25 °C. As *Neoscytalidium* grows faster than dermatophytes, cultures were positive in 2 to 4 days. Identification was performed with macro/microscopic observation of the culture.

### Onychomycosis cases

All skin and nail samples collected in our laboratory between January 1, 2010 and December 31, 2011 were reviewed to identify the cultures that led to the macro/microscopic identification of *Neoscytalidium* spp*.*

## Case presentation (Table 1)

**Table 1:** Characteristics of 5 kidney transplant recipients with invasive or disseminated *Neoscytalidium* infection

Patient	Age	Sex	Year of diagnosis	Origin	Time from transplantation (months)	Immunosuppressive regimensb	Type/Localization	Presence of skin appendage involvement	Identification	Treatment		Outcome
										First line	Second line	
1	53	Male	2001	French Guyana	3	Mycophenolic acid (250 mg), tacrolimus (6 mg) and prednisone (20 mg)	Deep cutaneous/right leg	Nail	*Neoscytalidium sp*	Itraconazole + surgical resection	Local amphotericin B added	Surgery first failed, then oral and local treatment succeeded after more than 4 months
2	64	Female	2002	Ivory Coast	134	Cyclosporine (100 mg) and prednisolone (10 mg)	Deep cutaneous/left foot	Right foot (sole and nail),	*Neoscytalidium hyalinum*	General (rapidly discontinued) and local terbinafine, 1 year		Clinical success, but samples positive 1 year after treatment
3	52	Male	2007	Mauritania	105	Mycophenolic acid (1500 mg), tacrolimus (3 mg) and prednisone (10 mg).	Deep cutaneous/right ankle		*Neoscytalidium dimidiatum*	Surgical resection only		Surgery succeeded
4	59	Male	2011	Cameroun	8	Mycophenolic acid (1000 mg), tacrolimus (30 mg), and prednisone (7.5 mg)	Disseminated: right leg and foot	Nail sample (2006)	*Neoscytalidium dimidiatum*	Voriconazole 200 mg bid	Local ketoconazole added	Resolved in 3 months, treatment 5 months.
5	49	Male	2011	Congo Brazzaville	15	Azathioprine (50 mg), tacrolimus (20 mg) and prednisone (15 mg)	Disseminated: cutaneous/sinal and pulmonary		*Neoscytalidium dimidiatum*	Voriconazole 200 mg bid		Resolved in 1 year

^a^For patients who underwent 2 transplantations, the time is the interval between diagnosis and the date of the second transplantation

^b^Immunosuppressive regimen at the time of diagnosis; dosages are per day

### Case 1

The first patient was a 53-year-old male who originated from French Guyana. He had received a kidney transplant in December 2000 for nephroangiosclerosis. At admission (in February 2001), his medications included mycophenolate mofetil 250 mg daily, tacrolimus 3 mg twice daily and prednisone 20 mg daily as immunosuppressant therapy. He was diagnosed as having Kaposi’s sarcoma with cutaneous, gastric and gut localizations. During his hospital work-up, a subcutaneous nodule was found on the right leg. A biopsy of the nodule demonstrated inflammation with fungal elements identified via mycological culture as *Neoscytalidium* spp. This same agent was found concomitantly on a toenail sample. Chest and abdomen CT scans for staging found no internal fungal localizations. The patient’s blood cultures were negative. A systemic oral antifungal therapy with itraconazole (400 mg twice daily) was initiated but 2 months later the leg lesion was still culture-positive. The lesion was thus excised but the surgery proved incomplete and the lesion remained secondarily positive. Thereafter, local treatments with amphotericin B-impregnated paraffin gauze were added, but again, samples collected 4 months after surgery were still positive for mycelium filaments and culture. The patient was then lost to follow-up for one and a half years. He reappeared in March 2005. Unexpectedly, no active fungal lesion was detected and he was therefore considered as cured of his *Neoscytalidium* infection.

### Case 2

The second patient was a 64-year-old female born in Ivory Coast. She was admitted for infection as a complication of Kaposi’s sarcoma in September 2002. Her medical history included polycystic renal disease that required renal transplantation in 1991, followed by extensive cutaneous Kaposi’s sarcoma in 1996. At admission, her medications included cyclosporine 100 mg daily and prednisolone 10 mg daily. During her hospitalization, various mycological examinations were positive: direct examination of left foot, right foot (sole and nail), and right leg lesion samples showed mycelian filaments identified as a *N. hyalinum* in culture. Magnetic resonance imaging of the left foot showed inflammatory and edematous remodeling of tissues, difficultly distinguishable from tumor infiltrations, but no bone lesions were observed. Initially, the treatment included both local and systemic terbinafine but an interaction with cyclosporine led to a fast deterioration of renal function. The systemic treatment was thus discontinued in regard to the risk for renal function and the clinical regression of the lesions. The local terbinafine treatment however was continued. Evolution was favorable with negative mycological cultures 4 months later.

### Case 3

The third patient was a 52-year-old male originating from Mauritania who had received an initial renal transplant in 1991 for polycystic renal disease and underwent a second renal transplantation in 2000. At admission, his medications included mycophenolate mofetil (750 mg twice daily), tacrolimus (3 mg daily) and prednisone (10 mg daily). In November 2007, he came back to the hospital for a post-traumatic lesion on the right leg. During the dermatological examination, an infiltrated, pigmented, and painless lesion measuring approximately 1.5 cm in diameter was found on the anterior face of the right ankle. A culture of the biopsy of this lesion grew *N. dimidiatum*. The lesion was resected successfully and no relapse was observed.

### Case 4

**Fig. 1:**
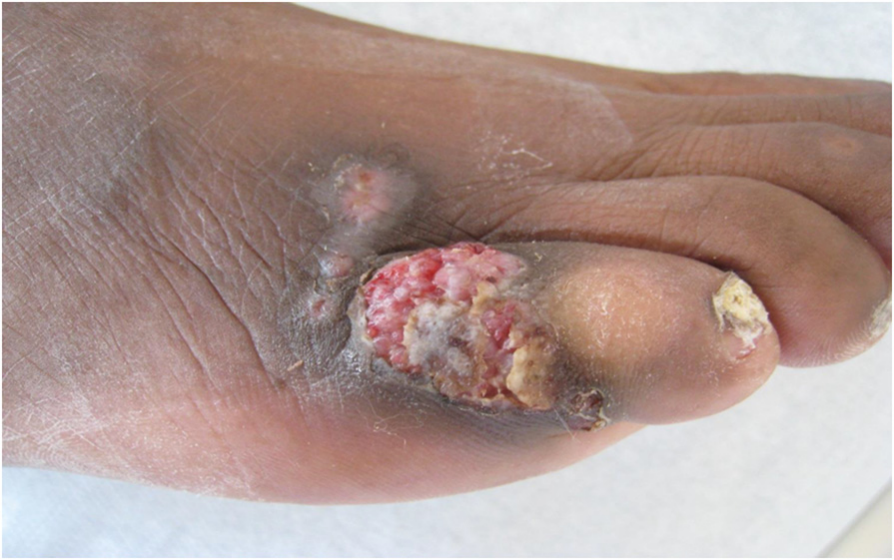
Lesion due to *Neoscytalidium dimidiatum* in a kidney transplant recipient

The fourth patient was a 59-year-old male originated from Cameroon admitted to the hospital in October 2011 for the exploration of two cutaneous lesions. He had undergone renal transplantation in 1992 for end stage renal disease of undetermined origin. He was receiving a second renal graft in February 2011. At admission, his treatment consisted of tacrolimus (15 mg twice daily), mycophenolate mofetil (500 mg twice daily) and prednisone (7.5 mg daily). One lesion in the right pretibial region and one ulcerative lesion on the dorsal surface of the fifth toe of the right foot measuring 1 × 2 cm were discovered during the dermatological examination (Fig. 1). These lesions had appeared 3 months earlier and grown progressively. Cultures of the lesions revealed *N. dimidiatum.* Histology of both lesions showed a granulomatous inflammation reaching the dermis. The diagnosis was thus a disseminated *N. dimidiatum* fungal infection with cutaneous involvement. Interestingly, *N. dimidiatum* had been previously identified on toenail samples in 2006. The patient was treated with voriconazole, 200 mg twice daily. The lesions regressed and remaining lesions were surgically excised and local treatment with topical ketoconazole applications under gauze was added. The lesions completely disappeared 3 months after the diagnosis and voriconazole was stopped after 5 months. No relapse occurred.

### Case 5

**Fig. 2:**
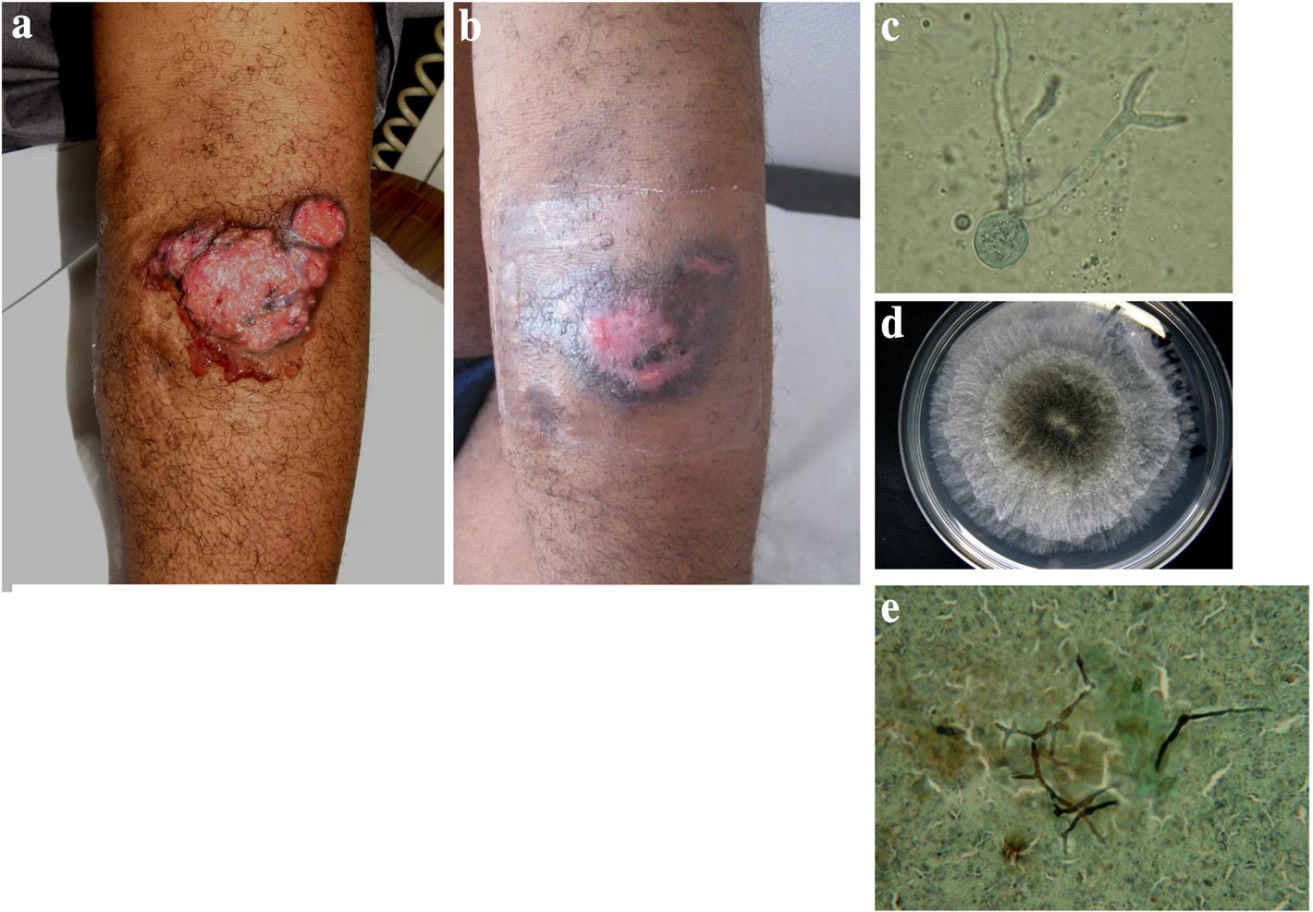
Disseminated *Neoscytalidium dimidiatum* infection in a kidney transplant recipient. Lesion on the left elbow at presentation (2**a**) and after 2 months of voriconazole therapy (2**b**). Other localizations included the lungs, sinuses and right leg (2**c** and 2**e**). Microscopic examination of the arm lesion showing hyphal fungal elements (2**d**). Photo of the 72-h culture from the arm lesion showing mold with a black center and white edges

The fifth patient was a 49-year-old male from the Republic of Congo. He was admitted to the hospital in May 2011 for multiple active cutaneous lesions. His medical history included a renal transplantation in February 2010 for a nephropathy of unknown origin. The lesions consisted of large painless ulcerated tumors on the left elbow, an infiltrated nodule on the back and an ulceration on the right leg (Fig. 2). Direct examination of samples from these lesions showed many fungal elements. Culturing led to the identification of *N. dimidiatum* at all sites. For that isolate, the macro/microscopic examination result was confirmed by internal transcribed spacer (ITS) sequencing using the following primers ITS1 5′-TCCGTAGGTGAACCTGCGG-3′ and ITS4 5′-TCCTCCGCTTATTGATATGC-3′. Moreover, bronchoalveolar lavage and nasal septum biopsy cultures were also positive, thus illustrating a disseminated form of *N. dimidiatum*. Blood cultures remained negative. Minimal inhibitory concentrations as determined by Etest (bioMérieux, Marcy l’Etoile, France) were: 0.032 μg/μL for voriconazole, 0.5 μg/μL for posaconazole, 12 μg/μL for fluconazole, and 0.38 μg/μL for amphotericin B. The minimal effective concentration was 0.064 μg/μL for both caspofungin and micafungin. Treatment with voriconazole was initiated and immunosuppression was reduced to a bitherapy with prednisolone and tacrolimus. Evolution was favorable with complete regression of the lesions (Fig. 2) within 12 months. The patient was then lost to follow-up for a year, but in the following year he was received for a medical visit during which the disappearance of lesions with residual scars and negative cultures were observed.

### *Neoscytalidium* superficial infections

In our hospital, over 2 years (January 1, 2010 to December 31, 2011), 4 466 cutaneous or skin appendage (i.e. nail, skin or hair samples) were obtained from 2 577 patients for fungal confirmation/identification, resulting in 1 540 (977 patients) positive cultures. Among these latter, *Neoscytalidium* spp*.* were identified in 77 isolates (5 % of all positive samples) from 42 patients representing 4.3 % of all patients with positive fungal culture. Precisely, 73.8 % of these *Neoscytalidium* patients (*n* = 31) had *N. dimidiatum* and 26.2 % (*n* = 11) *N. hyalinum*.

## Discussion

In endemic regions, *Neoscytalidium* spp. are an important cause of onychomycosis, accounting for 9 % of cases in Nigeria, 24 % in Gabon, and up to 56 % in the West Indies [23]. A retrospective study on cases occurring between 1994 and 1999 in mainland France showed that *Neoscytalidium* spp. infections accounted for 3.6 % of onychomycosis cases [3]. Similar results have been found in the United Kingdom where *Neoscytalidium* spp. were responsible for 3 % of onychomycosis cases [24]. These cases are imported, involving migrants from endemic regions [3]. The epidemiological data concerning *Neoscytalidium* superficial infection obtained in our hospital over a 2-year analysis period (January 1, 2010 to December 31, 2011) were consistent with those of previous epidemiological studies performed in Paris and London [25, 26] with respectively 67 and 73 % of cases of neoscytalidiosis due to *Neoscytalidium dimidiatum*.

Only a few cases of invasive/disseminated *Neoscytalidium* infections have been described. An aspect of interest for our case reports, although we only have five, is that they all occurred in renal transplant patients. In an article describing one case of invasive *N. dimidiatum* infection in an immunocompetent adult and reviewing 10 others cases of invasive *Neoscytalidium* infections with affection of deep organs, Elinav et al. reported that most of their invasive *Neoscytalidium* infections occurred in immunocompromised patients, including lung, renal or cardiac transplant recipients [11]. Additional aspects retrievable from the literature include other underlying conditions such as trauma, diabetes and cirrhosis. *Neoscytalidium* infections can occur in immunocompetent patients, with at least two cases reported. *Neoscytalidium* infections have been reported to manifest as central nervous system abscess, endophthalmitis, empyema, sinusitis and spondylodiscitis. Some authors have reported a poor prognosis with 50 % mortality for these infections but did not outline attributable mortality. Interestingly, of the deaths reported in the literature, two occurred in renal transplant patients [11–21, 27].

Deep localized subcutaneous infections (similar to what we report) are more frequently described in the literature. These lesions have been reported in immunocompromised patients using long-term corticosteroids [16, 28], in a cardiac transplant recipient [29], in renal transplant recipients [7, 30], in a discoid lupus erythematosus patient [31], in a case of liver cirrhosis [32], in a granulocytopenic child [21] and in a T-cell deficient patient [33]. Most of them were treated with a combination of systemic and local antifungal agents. Skin is more generally a frequent site of phaeohyphomycosis, up to 26 % in one review [34].

All of our patients were from the Caribbean overseas departments of France or from Africa and frequently returned to their places of origin where *Neoscytalidium* spp. are endemic [1]. Interestingly, one patient had a concomitant onychomycosis due to *Neoscytalidium* (patient 1) and another had been diagnosed for onychomycosis due to *Neoscytalidium* 5 years earlier (patient 4). Patient 3 had similar lesions at an interval of 6 years. Unfortunately, the fungus in the earlier lesion was not identified (culture remained sterile), which reveals the difficulties in diagnosing these infections. These observations suggest that patients with “dermatophyte-like” superficial infections with mild or no symptoms are at risk of developing a deep cutaneous infection after many years of immunodepression. Therefore, in patients who are particularly at risk, i.e. immunocompromised and originated from tropical zones, checking and even sampling for onychomycosis should be done.

We note that our cases occurred exclusively in renal transplant recipients despite the fact that our institution also performs heart, liver and hematopoietic stem cell transplantations. We do not have a specific explanation for this observation but it is likely that the limited number of cases may play a role and moreover impair a relevant statistical analysis. We note too that all our patients were severely immunocompromised and had been receiving immunosuppressive drugs for a very long time. Two had undergone renal transplantation twice. Furthermore, opportunistic infections (i.e. Kaposi’s sarcoma, *Pneumocystis jiroveci* pneumonia and toxoplasmosis) were diagnosed in three of our patients, reflecting the impairment of their immune function.

We encountered diagnostic difficulties in two patients with Kaposi’s sarcoma. In one, it was difficult to determine if the concerned lesions were due to secondary infection of Kaposi’s sarcoma lesions or primitive fungal infections, especially on the patient’s black skin. A link between Kaposi’s sarcoma and *Scytalidium* infection has been reported in HIV-infected patients [35]. This might suggest either that Kaposi’s lesions favor fungal infection or that fungal lesions may be misidentified and necessitate multiple biopsies and cultures for various infectious analyses.

*Neoscytalidium* spp. infections have no standardized treatment [1]. The mold is frequently resistant in vivo despite in vitro susceptibility tests showing low minimal inhibitory concentrations [MICs] [36–38]. In superficial infections, oral and topical treatments classically used against dermatophytes [griseofulvin, ketoconazole, itraconazole] are often ineffective with *Neoscytalidium* spp., possible due to melanin production [7, 36]. Successful eradication of mycosis has been obtained with systemic use of azoles [voriconazole and itraconazole] or with amphotericin B. However, in most cases, healing is protracted and combinations of drugs are often necessary [39].

In disseminated infections, many treatments have been used with various outcomes. Elinav et al. reported that four patients died and one was cured with amphotericin B, three died and one was cured with voriconazole, one died with posaconazole, and one was cured with ketoconazole [11].

In our series, all patients were cured. The difficulties in eradicating this infection and the absence of standardized care are reflected in the variety of treatments used for our patients. Importantly, patient five, who presented a disseminated infection and large cutaneous lesions, had a very good response under voriconazole only and no need for surgical resection of the lesions. It should be noted that the immunosuppressive regimen of this patient was downscaled, which might have enhanced his cure. In the other cases, the combination of surgical excision and medical treatment of lesions appeared to be a good option, as described in the literature, but we obtained successful outcomes as well with only oral voriconazole monotherapy in one patient and with only surgical excision for another.

## Conclusion

Deep cutaneous infections due to the black exotic mold *Neoscytalidium* spp. are rare events occurring primarily in immunocompromised patients such as the renal transplant recipients described here. Dissemination can occur and threaten prognosis. Systemic azole therapy appears to be of interest and may prevent the need for an immediate surgical intervention in some cases, although for others, surgery remains the most appropriate solution.

## Consent

All patients provided written informed consent for the publication of the case report and any accompanying images. A copy of the written consent is available for review by the Editor of this journal.

## Abbreviations

CT: Computed tomography
EORTC/MSG: European Organization for Research and Treatment of Cancer/Mycoses Study Group
ITS: Internal transcribed spacer
MICs: Minimal inhibitory concentrations
